# YOU ARE ONLY MY STUDENT FOR A LITTLE WHILE; YOU ARE MY COLLEAGUE FOR LIFE

**DOI:** 10.1093/geroni/igad104.0798

**Published:** 2023-12-21

**Authors:** Harvey Sterns

**Affiliations:** The University of Akron, Akron, Ohio, United States

## Abstract

The philosophy of you are my student for a little while-you are my colleague for life was shared with me by my major professor Paul Baltes. This has been my approach over my 50+ year career. I have had a number of significant mentors and have had many great students. In the spirit of this award, I will share my understanding of mentorship.

